# Inorganic Phosphate (Pi) Signaling in Endothelial Cells: A Molecular Basis for Generation of Endothelial Microvesicles in Uraemic Cardiovascular Disease

**DOI:** 10.3390/ijms21196993

**Published:** 2020-09-23

**Authors:** Nima Abbasian, Alan Bevington, James O. Burton, Karl E. Herbert, Alison H. Goodall, Nigel J. Brunskill

**Affiliations:** 1 Department of Cardiovascular Sciences, University of Leicester, and Leicester NIHR Cardiovascular Biomedical Research Unit, Leicester LE3 9QP, UK; jb343@le.ac.uk (J.O.B.); keh3@le.ac.uk (K.E.H.); ahg5@le.ac.uk (A.H.G.); njb18@le.ac.uk (N.J.B.); 2 Department of Nephrology, Leicester General Hospital, Leicester LE5 4PW, UK

**Keywords:** inorganic phosphate, PP2A, Src, DAPK-1, tropomyosin, signaling, microvesicles

## Abstract

Hyperphosphataemia increases cardiovascular mortality in patients with kidney disease. Direct effects of high inorganic phosphate (Pi) concentrations have previously been demonstrated on endothelial cells (ECs), including generation of procoagulant endothelial microvesicles (MVs). However, no mechanism directly sensing elevated intracellular Pi has ever been described in mammalian cells. Here, we investigated the hypothesis that direct inhibition by Pi of the phosphoprotein phosphatase PP2A fulfils this sensing role in ECs, culminating in cytoskeleton disruption and MV generation. ECs were treated with control (1 mM [Pi]) vs. high (2.5 mM [Pi]), a condition that drives actin stress fibre depletion and MV generation demonstrated by confocal microscopy of F-actin and NanoSight Nanoparticle tracking, respectively. Immuno-blotting demonstrated that high Pi increased p-Src, p-PP2A-C and p-DAPK-1 and decreased p-TPM-3. Pi at 100 μM directly inhibited PP2A catalytic activity. Inhibition of PP2A enhanced inhibitory phosphorylation of DAPK-1, leading to hypophosphorylation of Tropomyosin-3 at S284 and MV generation. p-Src is known to perform inhibitory phosphorylation on DAPK-1 but also on PP2A-C. However, PP2A-C can itself dephosphorylate (and therefore inhibit) p-Src. The direct inhibition of PP2A-C by Pi is, therefore, amplified by the feedback loop between PP2A-C and p-Src, resulting in further PP2A-C inhibition. These data demonstrated that PP2A/Src acts as a potent sensor and amplifier of Pi signals which can further signal through DAPK-1/Tropomyosin-3 to generate cytoskeleton disruption and generation of potentially pathological MVs.

## 1. Introduction

Phosphate compounds play a pivotal role in all cells, in processes as disparate as energy homeostasis; nucleotide, nucleic acid and phospholipid metabolism and signaling through protein phosphorylation events. Recently renewed interest in the functional and pathological effect of orthophosphate (Pi) has been driven by evidence that elevated serum Pi concentrations are strongly associated with an increased risk of cardiovascular events and mortality, particularly in patients with hyperphosphataemia, a hallmark of chronic kidney disease (CKD) [1], but also in the general population with normal renal function and higher serum Pi concentration, although within the normal range [2,3,4]. It has been also reported that in vivo Pi is associated with other major pathological processes, including renal fibrosis [5], angiogenesis [6], carcinogenesis [7] and tumour progression [8]. Furthermore, in vitro profound effects of physiologically relevant Pi concentrations have been demonstrated on endothelium, including endothelial–mesenchymal transition [9] and generation of procoagulant endothelial microvesicles (MVs) [10].

Even though mechanisms for the sensing of [Pi] in bacteria, yeast [11,12] and plants [13] have been described in detail, and a cell surface sensing of Pi by plasma membrane phosphate transporter proteins (SLC20; solute carrier Pi transporter) has recently been proposed in mammalian cells [14], no molecular mechanism directly sensing and propagating a signal following a rise in the intracellular Pi concentration has been described in mammalian cells.

Raising extracellular Pi concentration can increase intracellular Pi in mammalian cells, typically within 1.5 h [10,15,16]. In human endothelial cells (ECs), this occurs by transport through active Na^+^-linked PiT1 (SLC20A1) Pi transporters, allowing Pi to act on intracellular phosphatases and subsequently induces a global increase in both protein Tyr and Ser/Thr phosphorylation [10].

**Figure 1:**
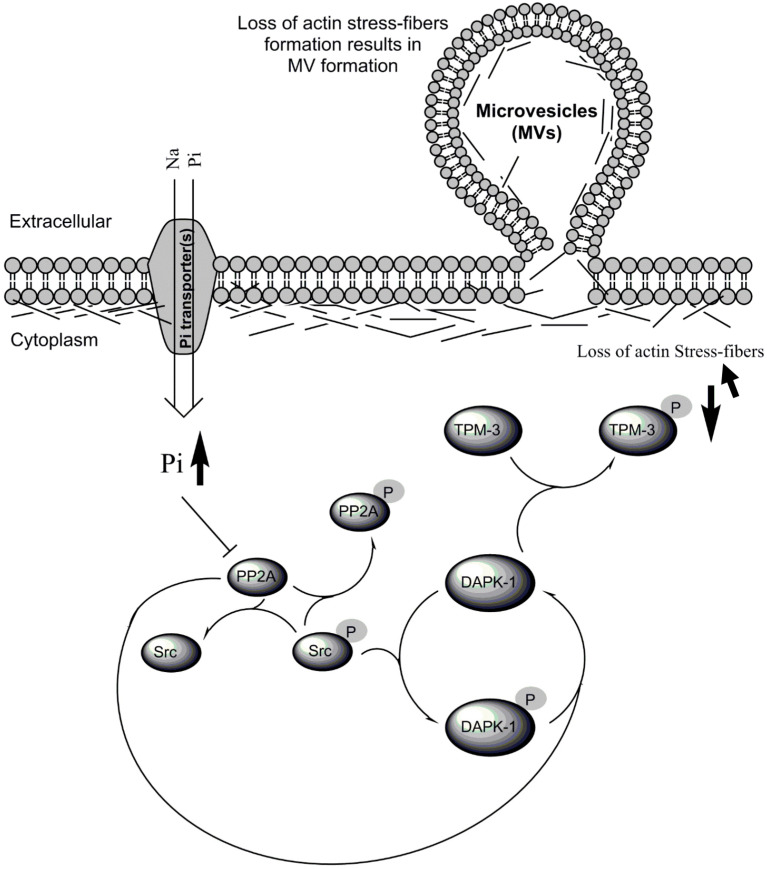
Diagram of proposed Pi-induced PP2A/Src/DAPK-1/TPM-3 signaling pathway. While hyperphosphorylation of TPM-3 (as the supporting link between TMP-3 and the cytoskeleton) [31] is associated with an enhanced actin stress-fibre formation and works as a protective mechanism in maintaining cell membrane integrity, hypophosphorylation of TPM-3 may result in loss of actin stress-fibres and be associated with membrane blebbing/MV generation from the cells. NB: Pi transporters include Na^+^-Linked PiT-1 (slc20a1) and PiT-2 (slc20a2), with the former the main Pi transporters in endothelial cells (ECs) (described in detail in the Introduction).

A number of phosphoprotein phosphatases have been reported to be directly inhibited by physiological Pi concentrations [17,18,19]. Phosphoprotein phosphatase PP2A consists of a large family of holoenzymes that accounts for about 1% of total cellular proteins and conveys the majority of Ser/Thr phosphatase activity in eukaryotic cells [20]. PP2A is ubiquitously expressed and is involved in the regulation of a wide range of cellular signaling and responses, notably neural development; Akt, NF-kB and MAPK signaling, apoptosis, and cell cycle progression [20]. Of particular relevance to the present study, PP2A has been implicated in the indirect inactivation of the nonreceptor tyrosine kinase Src [21] (Figure 1), and the dephosphorylation of death-associated protein kinase 1 (DAPK-1) [22,23] (Figure 1).

Src plays a vital role in diverse cellular processes, including cell proliferation, survival and drug resistance [24,25,26]. Src tyrosine kinase activity can be regulated through the control of its phosphorylation status by kinases and phosphatases. Dephosphorylation of its carboxy-terminal Tyr-530 and autophosphorylation on Tyr-419 activates this enzyme [27]. There is evidence indicating that Src activation results in inhibitory phosphorylation on DAPK-1 [28] and also PP2A by inducing phosphorylation on the catalytic subunit of PP2A (PP2A-C) [25,29]. The inhibitory phosphorylation of PP2A-C inhibits its phosphatase activity. As noted above, PP2A-C can itself inhibit Src [26], so the mutual effects of PP2A-C and Src on one another may allow the initial direct inhibitory effect of Pi on PP2A-C to be amplified (Figure 1).

Phosphorylation of DAPK-1 has been shown to reduce the kinase activity of this enzyme [28,30], and subsequent proteasomal degradation of the phosphorylated DAPK-1 protein reduces the kinase activity of this enzyme even further. On the other hand, DAPK-1 is known to phosphorylate cytoskeletal regulatory protein Tropomyosin [31], and this phosphorylation has been shown to serve as a protective mechanism, keeping the integrity of the cell membrane. Conversely, previous evidence [31] suggests that hypophosphorylation of Tropomyosin (which might be triggered by Pi-induced PP2A inhibition (Figure 1)), would result in the loss of actin stress fibre formation and an associated increase in membrane blebbing and MV generation [31] (Figure 1). In this study, we tested the hypothesis (Figure 1) that direct inhibition of PP2A by Pi can fulfil such a sensing role (i.e., direct sensing of a signal following a rise in intracellular [Pi]) in endothelial cells, with subsequent positive feedback propagation of the signal through the protein kinase Src. We further demonstrated how this signal is subsequently directed to the cytoskeletal regulatory protein Tropomyosin-3 (TPM-3), where it may play a vital role in actin cytoskeleton rearrangement resulting in the generation of MV.

## 2. Results

### 2.1. Pi Directly Inhibits PP2A Catalytic Activity

**Figure 2:**
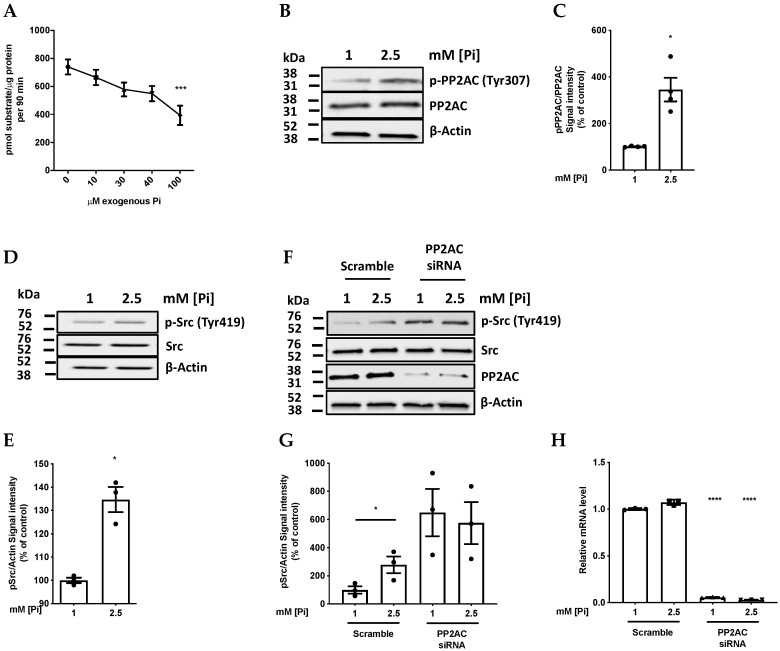
Pi inhibits PP2A and induces an increase in Src phosphorylation. (**A**) Direct inhibition by Pi of PP2A catalytic activity after pulling-down endogenous PP2A-C from EA.hy926 ECs lysates and subjecting this to phosphatase catalytic activity assay in the presence of exogenous Pi at the stated concentration using PP2A phosphatase substrate (Millipore Ref 17-313). *n* = 3, *** *p* = 0.0002 versus control without Pi. (**B**) Representative immunoblot demonstrating Pi-induced inhibitory phosphorylation of PP2A in intact EA.hy926 cells. Effect of 1.5 h Pi-loading hyperphosphatemia on expression of PP2AC and p-PP2AC (Tyr307). (**C**) Densitometry analysis of (**B**), *n* = 3, * *p* = 0.037. (**D**) Representative p-Src immunoblots probed using anti-p-Src (Tyr419) antibody and (**E**) corresponding densitometry analysis of (**D**) *n* = 3, * *p* = 0.023. (**F**) Representative immunoblots and (**G**) quantitative analysis by densitometry of the effect of siRNA silencing of PP2A-C on Src phosphorylation during 1.5 h incubations of cells with 1 or 2.5 mM Pi. *n* = 3, * *p* = 0.0337. (**H**) Relative mRNA levels of PP2A-C in EA.hy926 cells transfected with scrambled siRNA and PP2A-C silencing siRNA for 1.5 h. After removal of the transfection medium and allowing an additional 24-h recovery period in growth medium, cultures were treated with 1 or 2.5 mM Pi, and RNA was extracted from the cells, reverse transcribed, and subjected to quantitative RT-PCR. *n* = 3, **** *p* < 0.0001.

The direct inhibitory effect of Pi on PP2A-C proposed in Figure 1 was tested first by pulling-down endogenous PP2A-C from cell lysates by immunoprecipitation, and subjecting this to colorimetric phosphatase catalytic activity assay. The inhibitory effect of Pi on PP2A phosphatase from EA.hy926 ECs was clearly detected with as little as 100 µM Pi (Figure 2A).

If this inhibition also occurs in intact cells, a secondary (Src-mediated) inhibitory phosphorylation of PP2A would be predicted (Figure 1). By probing cell lysates with antibody recognising phospho-PP2A-C (Tyr307), it was shown that adding 2.5 mM Pi to intact cultures of EA.hy926 cells for as little as 1.5 h did indeed increase this inhibitory phosphorylation of PP2A-C, yielding a 3-fold increase relative to control cultures with 1 mM physiologically normal Pi (Figure 2B,C).

### 2.2. High Pi Activates Src and PP2A-C Partial Silencing Preserves Src Phosphorylation

To demonstrate that the Pi-induced promotion of PP2A-C phosphorylation (and hence inhibition of its activity) was giving the predicted phospho-activation of Src, Western blotting was performed using an antibody against p-Src (Tyr419). This showed that 2.5 mM extracellular [Pi] acutely induced hyperphosphorylation of Src at Tyr419 within 1.5 h in cultures of EA.hy926 ECs (Figure 2D,E). To confirm that this was mediated by PP2A inhibition, siRNA silencing of PP2A-C gene expression was performed using an siRNA which efficiently knocked down PP2A-C expression both at the protein level (Figure 2F,G) and at the mRNA level (Figure 2H). This strongly increased p-Src (Tyr419), indicating that in these cells, this phosphatase does exert the previously described [21] indirect stimulatory effect on Src phosphorylation (Figure 2F). As expected, silencing of PP2A-C expression abolished the effect of Pi on Src phosphorylation but, in the presence of an irrelevant scrambled siRNA sequence, a significant (~3-fold) Pi-induced stimulation of Src phosphorylation was still observed (Figure 2F).

### 2.3. High Pi Inactivates DAPK-1 by Inducing Phosphorylation at Ser 308

**Figure 3:**
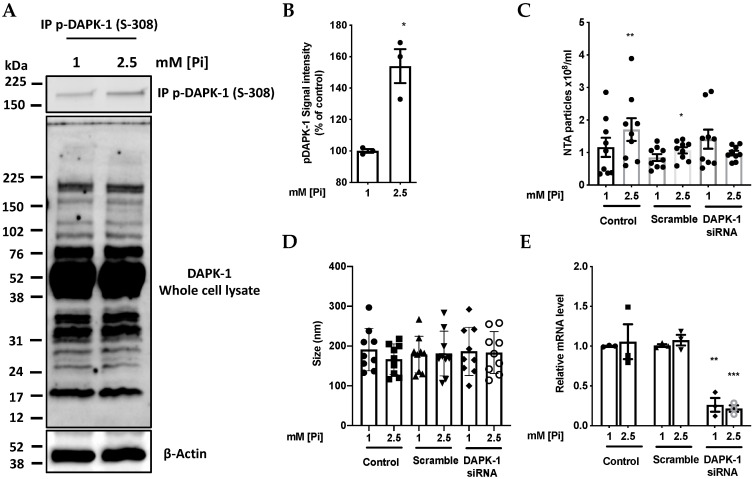
Pi induces an increase in phosphorylation of DAPK-1. (**A**) Endogenous human DAPK-1 was immuno-precipitated from cell lysates (200 µg total cellular protein) from cells treated with 1 or 2.5 mM Pi for 1.5 h. Immuno-precipitated DAPK-1 was subjected to immuno-blotting with phosphospecific antibody against p-(S308)-DAPK-1. (**B**) Densitometry analysis of (**A**), *n* = 3, * *p* = 0.037. (**C**) Partial silencing of DAPK-1 blunts the effect of elevated Pi concentration on MV release detected by NanoSight Nanoparticle Tracking Analysis (NTA). Each pair of data points refer to a separate multiwall plate, *n* = 4, * *p* = 0.015, ** *p* = 0.0035, (**D**) Size of particles released from cultures in response to Pi-loading for 1.5 h as in (**C**) measured by NTA. (**E**) Relative mRNA level of DAPK-1 confirming siRNA silencing of DAPK-1as indicated in (**B**–**D**), *** *p* = 0.0002.

To show that these Pi-induced changes in PP2A and Src were leading to functionally important downstream signals culminating in increased MV output, the resulting effects on DAPK-1 were studied. From the scheme in Figure 1, PP2A inhibition and Src activation would both be expected to increase DAPK-1 phosphorylation. The predicted high Pi-induced inhibitory phosphorylation of DAPK-1 on Ser308 was indeed observed (Figure 3A,B), and was accompanied by a significant increase in MV output from the cultures (Figure 3C,D), as we have reported previously [10]. As expected, efficient siRNA silencing of DAPK-1 expression using DAPK1 Select validated siRNA (Figure 3E) completely abolished this effect of Pi on MV output (Figure 3C).

### 2.4. High Pi induces Hypophosphorylation of TPM-3 on Ser284

**Figure 4:**
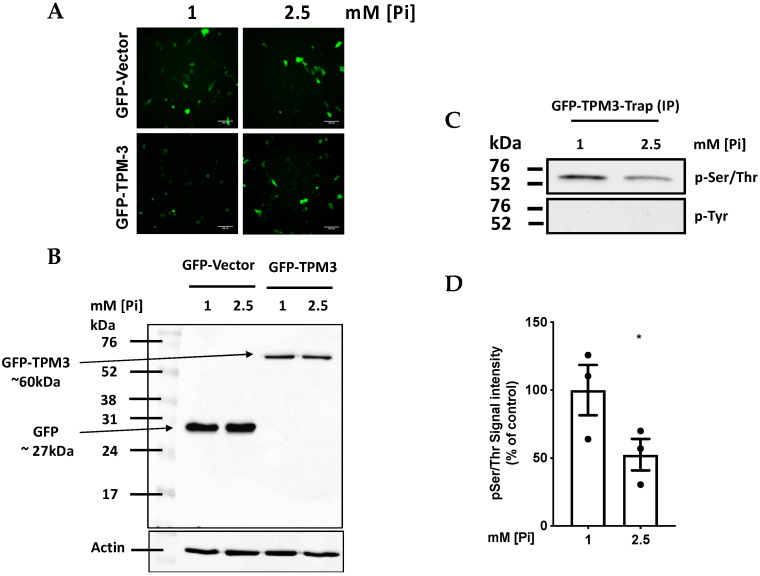
High Pi induces hypophosphorylation of TPM-3 on a Ser/Thre residue. ECs were transfected with empty GFP-vector or GFP-tagged TPM-3. Thereafter cells were treated with 1 vs. 2.5 mM [Pi] for 1.5 h. (**A**) Fluorescence microscopy confirming that the cells were transfected successfully. (**B**) Immuno-blotting using anti-GFP antibody confirming the expression of free GFP (lanes 1 and 2) and TPM-3-GFP (lanes 3 and 4). (**C**) Immuno-blotting using pan-specific antibodies against pSer/Thr (upper panel) and pTyr (lower panel) demonstrating that GFP-trapped TPM-3 from 200 µg total cellular protein is hypophosphorylated on Ser/Thr but not Tyr in cells treated with 2.5 mM Pi (**D**) densitometry analysis of (**C**), *n* = 3, * *p* = 0.033.

The data on DAPK-1 silencing imply that this kinase is acting on a target protein which plays an important role in cytoskeleton function and hence MV output. The cytoskeletal regulatory protein TPM-3 (which is the predominant Tropomyosin isoform expressed in EA.hy926 cells [10]) has 7 Serine (Ser) and 3 Threonine (Thr) residues that could potentially be phosphorylated by the serine-threonine kinase DAPK-1, and hence show hypophosphorylation when DAPK-1 is inhibited. To investigate the hypothesis that Pi induces hyphophosphorylation of TPM-3, ECs were transfected with plasmids expressing GFP-tagged wild-type TPM-3 or control GFP plasmid. Transfected cells were then treated with 1 or 2.5 mM [Pi] for 1.5 h, and then the expression of free GFP or the GFP-TPM-3 fusion protein was confirmed by fluorescence microscopy (Figure 4A) and by Western blotting with anti-GFP antibody (Figure 4B). The GFP-TPM-3 fusion protein was then pulled-down from cell lysates by GFP-Trap^®^. Western blotting of the GFP-Trapped proteins using pan-specific antibodies against phospho-Ser/Thr and phospho-Tyr demonstrated Serine and/or Threonine phosphorylation within the GFP-TPM-3 protein, and reproducible hypophosphorylation under high Pi conditions (Figure 4C,D). No Tyr phosphorylation was detected.

**Figure 5:**
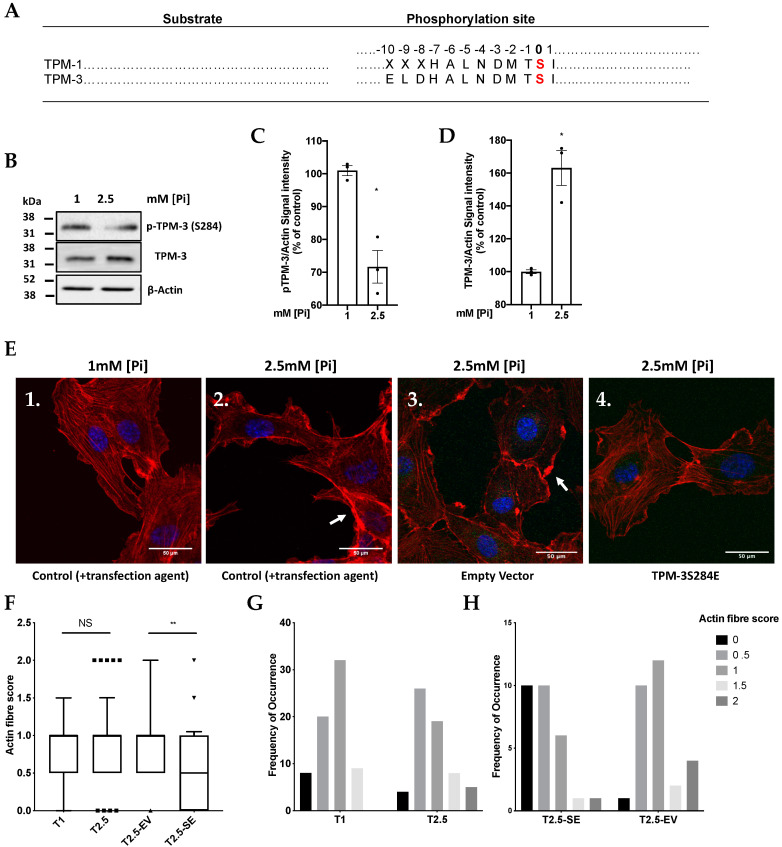
Pi-induced hypophosphorylation of cytoskeletal regulatory protein (TPM-3) resulting in the loss of actin stress-fibre formation which is reversed by overexpression of pseudophosphorylated TPM-3 mutant. (**A**) Sequence alignment of human TPM-1 and TPM-3 showing the amino acid sequence around the phosphorylation site of TPM-3 at the C-terminus. (**B**) p-TPM-3 (S284) and total TPM-3 representative immunoblots obtained from cell lysates using antibodies against TPM-3, and p-(S284)-TPM-3 after treatment of the cells with 1 or 2.5 mM Pi for 1.5 h. (**C**,**D**) Blots were quantified by densitometry (*n* = 3, * *p* = 0.0337, ** *p* = 0.0095). (**E**) High Pi-induced hypophosphorylation of TPM-3 after treating the cells with 1 or 2.5 mM [Pi] for 1.5 h is associated with the loss of actin stress fibre formation, but accumulation of cortical actin deposits (Panel 2). In contrast, cultures transfected with plasmid overexpressing pseudophosphorylated TPM-3 mutant (TPM-3S284E) show partial restoration of stress fibres in the presence of 2.5 mM Pi (Panel 4). After cells were treated with 1 or 2.5 mM [Pi] for 1.5 h, they were fixed and stained for F-actin using Alexa-Fluor-594-phalloidin and DAPI and then were examined by confocal microscopy. White arrows show formation of dense actin deposits in the cortical region of the cell. (**F**–**H**) A quantification from the above experiments was performed (*n* = 3, ** *p* = 0.0021) as detailed in the experimental procedures section. SE: pseudophosphorylated TPM-3 mutant, EV: Empty Vector, NS: Not significant. Actin fibre score data in (**F**) are presented as box and whisker plots.

An earlier study on Tropomyosin-1 (TPM-1) [31] showed phosphorylation by DAPK-1 on Ser residue 283. Alignment of the TPM-1 and TPM-3 sequences (Figure 5A) suggested that DAPK-1 phosphorylation of TPM-3 might, therefore, occur on Ser 284. To confirm this, a custom phospho-specific antibody recognising TPM-3 (Ser284) was raised and used to probe lysates from cells treated with 1 and 2.5 mM [Pi] for 1.5 h. In agreement with Figure 4C, this showed that high Pi induces hypophosphorylation of TPM-3 (Ser284) (Figure 5B,C), accompanied by a detectable increase in total TPM-3 (in agreement with previous findings from this laboratory [10]), resulting in a marked decline in the p-TPM-3/Total TPM-3 ratio (Figure 5B,D).

### 2.5. Pi and TPM-3 Phosphorylation Alters Actin Stress-Fibre Organisation within ECs

Finally, to demonstrate a functional link between the Pi effects on DAPK-1 phosphorylation and MV output in Figure 3, the Pi effects on TPM-3 phosphorylation in Figure 4 and Figure 5B and the structure of the actin cytoskeleton, the effect of high Pi loaded medium on stress fibre formation was investigated by performing F-actin staining of the actin cytoskeleton in wild-type and phosphorylation mimicking mutant of TPM-3 generated by site-directed mutation of TPM-3 C-terminus from Ser284 (TCT) to pseudophosphorylated Glu284 (GAA) (Supplementary Figure S2). Looking at the frequency distribution of the actin fibre scores (blind-scored (as previously described)) [31] (Figure 5F,G), treating cells with 2.5 mM [Pi] for 1.5 h reduced actin stress fibres in endothelial cells, accompanied by accumulation of thick cortical actin deposits under the plasma membrane on the edge of the cells or in thick dense actin fibres (Figure 5E—Panel 2 and 3). It is worth noting that a high score of “2” attributable to cells expressing dense actin deposits on the edge of the cells or in thick dense actin fibres was never recorded in endothelial cells treated with 1 mM [Pi] (Figure 5E—Panel 1). Transfecting endothelial cells with a Ser284Glu mutant of TPM-3 also seemed to skew the scores in Pi-loaded cultures back towards lower scoring, indicating that the loss of stress fibre formation (accompanied by TPM-3 hypophosphorylation (Figure 4 and Figure 5B)) was reversed by expressing the pseudophosphorylated TPM-3 mutant Ser284Glu (Figure 5E—Panel 4). This suggests that (in agreement with Figure 3) the Pi signals through PP2A/Src to DAPK-1 resulting in TMP-3 hypophosphorylation led to the predicted changes in the cytoskeleton and hence MV output.

## 3. Discussion

### 3.1. Pi Sensing through Signaling from PP2A to Src

In this study, we provide evidence for the first time that an elevated intracellular Pi concentration can be sensed directly in a mammalian cell line by the Pi-responsive protein phosphatase PP2A.

An important feature of the proposed mechanism (Figure 1) is the concept that, following inhibition of its catalytic activity by elevated Pi, PP2A is able to signal to Src, culminating in autophosphorylation of the activated Src kinase on Tyr-419 (Figure 2D–H). Such linkage between PP2A and Src has also been reported elsewhere [21,26]. As PP2A normally functions as a phospho-serine-threonine phosphatase, it is unlikely that this arises from the direct action of PP2A on the Tyr-419 phosphorylation site, although Tyr phosphatase activity has been ascribed to PP2A under some circumstances [32].

### 3.2. The Need for Intracellular Amplification in the Sensing of Extracellular Pi by Cells

Apart from a recent interesting study on plasma membrane Pi transporter proteins [14], no sensor has ever been described on the surface of mammalian cells which is capable of responding to changes in the extracellular Pi concentration. This implies that at least part of the sensing of ambient Pi concentration occurs inside cells following increased Pi transport through the plasma membrane (Figure 1), resulting in a rise in the intracellular Pi concentration. However, in the EA.hy cells studied here, 1.5 h of exposure to a 2.5-fold increase in the extracellular Pi concentration typically only results in an increase in the order of 50% in the intracellular Pi concentration [10]. The fractional increase may be even smaller in other cultured cell lines [16], and a limited response is also observed when free cytosolic Pi (rather than total Pi) is measured by the use of 31P nuclear magnetic resonance spectroscopy [33,34]. Cells which show a significant biological response to Pi are, therefore, likely to be responding to relatively modest elevations in the intracellular Pi concentration which are then amplified in some way to generate a substantial downstream signal. This may explain why the initial response to Pi described here in EA.hy926 cells is not only a direct inhibitory effect of Pi on PP2A catalytic activity (Figure 2A) but also a reinforcing feedback effect of Src on PP2A, leading to further PP2A inhibition through the inhibitory phosphorylation of PP2A on Tyr 307. The net effect is that a modest rise in intracellular Pi results in a 3-fold increase in PP2A phosphorylation (Figure 2B,C).

### 3.3. Pi Sensing by Proteins Other Than PP2A

It is important to emphasise that the evidence presented here is not intended to exclude the possibility that Pi is also sensed by proteins other than PP2A—in EA.hy926 cells or in other mammalian cell types. At least in vitro, physiologically attainable Pi concentrations have been shown to activate or inhibit the biological activity of a number of important regulatory proteins, including phosphoprotein tyrosine phosphatases (PTPs) [17,18,19] and a recent report of transport-independent effects on SLC20 Pi transporters [14]. Of particular interest, for the future investigation of intracellular Pi signals are phospho-tyrosine phosphatases such as PTP1B, which is inhibited by the structural analogue of Pi, orthovanadate [35], particularly in view of the previous observation from this laboratory that orthovanadate can acutely mimic the stimulatory effect of Pi on MV output in EA.hy926 cells [10]. However, in view of the abundant evidence that PTP1B is the dominant phosphatase responsible for the removal of the inhibitory phosphorylation at Tyr-307 on PP2A-C [36,37,38], it is very likely that putative PTP1B inhibition by Pi feeds directly into the pathway described here, supplementing the action of Src that is responsible for the potent Pi-induced hyperphosphorylation of PP2A-C that was observed in Figure 2B.

### 3.4. Biological Significance of Pi Signaling through PP2A/Src to DAPK-1

It has long been suspected that Pi exerts important “Pi toxicity” [1] effects on cells in vivo, resulting in serious pathological effects, especially in the cardiovascular system [1,2,3,4]. However, a major limitation in studying this problem in vivo has been a lack of suitable molecular markers for phosphate toxicity or for the Pi-induced signaling pathways within cells that lead to it. In principle, elevation of the intracellular Pi concentration could be taken as a direct indicator of such effects, but this is technically difficult to measure in vivo because of artefactual increases driven by ATP catabolism to Pi during harvesting of tissue, or artefactual chemical decomposition of organic phosphates to Pi during sample processing or assay. The data presented in this paper now show protein phosphorylation markers directly responsive to elevated intracellular Pi concentration, which are potentially of wide relevance in view of the ubiquitous expression of the proteins involved (PP2A and Src) in mammalian cells. The signal beyond Pi through PP2A/Src to DAPK-1 is also of wider significance in view of the fundamental role of DAPK-1 in apoptosis, autophagy and membrane blebbing [39], particularly in hypoxic or ischaemic tissues in which elevation of intracellular Pi as a result of declining energy status [40], therefore, provides a potentially important link between hypoxia, ischaemia and death signaling.

The data presented in Figure 5 with the TPM-3 mutant S284E suggest that a key molecular event affecting the actin cytoskeleton is the phosphorylation status of S284 in TPM-3, in agreement with an earlier study on the closely similar protein TPM-1 in endothelial cells, in which the corresponding serine residue is S283 [31]. In principle, the biological effect of phosphorylation of S284 in TPM-3 could also be demonstrated by an alanine mutant S284A, provided that such a mutant (incapable of being phosphorylated) exerts a “dominant negative” effect, i.e., negates the biological effect of the phosphorylated S284 wild-type TPM-3. This possibility was tested in the previous study on TPM-1 [31]. However, even though apparent changes were seen in the morphology of the actin cytoskeleleton in some cells in response to transfecting with an S283A mutant, careful quantification by those authors detected no reproducible quantitative effect (Figure 5 in [31].) In view of this apparent absence of a biological effect of the serine to alanine mutation, the present study was confined to the phospho-mimetic S284E mutant.

In conclusion, this study shows how, in endothelial cells, DAPK-1 and TPM proteins downstream from PP2A/Src can give functionally important cytoskeletal rearrangements and output of MVs, which, as we have shown previously [10], are procoagulant, and hence may contribute to clinically significant thrombotic events. For that reason, it will now be of considerable interest to investigate the occurrence of this pathway from Pi to DAPK-1 and TPM in endothelium during hyperphosphataemia in vivo, especially in CKD in which the accumulation of procoagulant endothelial MVs is already known to be an important feature of uraemic hyperphosphataemia [41].

## 4. Materials and Methods

### 4.1. Cell Culture and Incubations

Immortalized human EC line EA.hy926 was used for all experiments between passages 5 and 20. Cells were maintained in DMEM (Thermo Fisher Scientific, Loughborough, UK) with 10% (vol/vol) heat-inactivated FBS, 2 mM L-glutamine, penicillin (10^2^ IU·mL^−1^) and streptomycin (100 mg·mL^−1^) at 37 °C in a humidified 5% CO2 atmosphere. Unless otherwise stated, cells were seeded for experiments in 35-mm six-well plates at 3 × 10^5^ cells/cm2 and used at 70% confluence. Experimental incubations were performed in MEM (Thermo Fisher Scientific, Loughborough, UK) with 2 mM L-glutamine, penicillin (10^2^ IU·mL^−1^) and streptomycin (100 mg·mL^−1^) at pH 7.4 with 1.8 mM [Ca2^+^] and 1 mM [Pi]. To model hyperphosphatemia, NaH_2_PO_4_ was added to raise the [Pi] to 2.5 Mm—a concentration that has been used extensively elsewhere [10,42,43,44]. Occasionally, cells were tested by a LookOut^®^ Mycoplasma PCR Detection Kit (Sigma-Aldrich, Gillingham, UK) according to the manufacturer’s instructions to confirm the absence of mycoplasma contamination.

### 4.2. Nanoparticle Tracking Analysis (NTA)

The number and size of the particles in medium harvested from cells were analysed by Nanoparticle Tracking Analysis (NTA) using a NanoSight LM10 with NTA software v2.2 (NanoSight Ltd., Amesbury, UK) and 90 s video capture as previously described [10,41].

### 4.3. Plasmids and siRNA

TPM-3 cDNA was cloned by PCR amplification from an ORF clone (SourceBioscience, Cambridge, UK: GC-D0474-10) into pEGFP-C1 vector using the following primers: 5′-GTATTTTCAGGGCGCCATGATGGAGGCCATCAAGAAAAA-3′, and 5′-GACGGAGCTCGAATTTCATTATATAGAGGTCATGTCATT-3′. The pseudophosphorylated TPM-3 mutant S284Glu was generated by PCR site-directed mutagenesis on a pEGFP-C1-TPM-3 construct using the primers 5′-ACGCCCTCAATGACATGACCGAAATATAA-3 and 5′-TTATATTTCGGTCATGTCATTGAGGGCGT-3′. The resulting plasmids were subjected to DNA sequencing to confirm the insertion of site-directed mutagenesis (Figure 5G). Plasmid transfection (1µg DNA per 35 mm culture) in EA.hy926 cells was performed using TransIT^®^-2020 Transfection Reagent (Geneflow, Staffordshire, UK).

Transient silencing of DAPK-1 and/or PP2A-C in cells was performed by siRNA targeting the specific genes using Silencer^®^ Select Validated siRNAs (Thermo Fisher Scientific, Loughborough, UK) with a scrambled nontarget Silencer^®^ Select Negative Control siRNA (Thermo Fisher Scientific, Loughborough, UK) as a negative control. siRNA oligonucleotides were incubated for 4 h with the cultures at 50 and 10 nM final concentration for DAPK-1 and PP2A-C, respectively, as described in the manufacturer’s instructions using Lipofectamine^®^ RNAiMAX Transfection Reagent (Thermo Fisher Scientific, Loughborough, UK). Uptake of siRNA was confirmed using BLOCK-iTTM Fluorescent oligonucleotides (Thermo Fisher Scientific, Loughborough, UK) followed by quantification by flow cytometry.

### 4.4. RNA Extraction and qRT-PCR

Total RNA was extracted using an miRNeasy Mini Kit (Qiagen, Manchester, UK). Using 1 µg total RNA, cDNA was synthesised using an AMV Reverse Transcription System (Promega, Hampshire, UK) according to the manufacturer’s instructions. Real-time PCR was performed using an Applied Biosystems 7500 Fast Real-Time PCR System (Applied Biosystems, Life Technologies, Carlsbad, CA, USA) using TaqMan^®^ Gene Expression Assay (Thermo Fisher Scientific, Loughborough, UK) for DAPK-1, PP2AC and 18S. Relative amounts of mRNA were normalised to the corresponding 18S signal for each sample, and relative expression is presented as 2^−ΔΔ*C*t^ [45].

### 4.5. Immunoblotting

Cell lysates were subjected to SDS-PAGE (20 µg protein per lane) followed by immunoblotting. Immunoblotting was performed onto nitrocellulose membranes (Amersham, Thermo Fisher Scientific, Loughborough, UK) followed by probing with primary antibodies against TPM-3 (Cell Signaling Technology, London, UK), global P-Tyr (Santa Cruz Biotechnology, Dallas, TX, U.S.A.), global P-Ser/Thr (Antibodies-online, BD), DAPK-1 (Sigma), p-DAPK-1 (Sigma), p-TPM-3 (Custom phospho-specific antibody, EUROGENTEC LTD, Liège, Belgium), Src (Cell Signaling), p-Src (R&D), PP2A-C (Cell Signaling), p-PP2A-C (Santa Cruz), GFP (Cell Signaling) and β-actin (Abcam, Cambridge, UK). Polyclonal Rabbit Anti-Mouse and Goat Anti-Rabbit Immunoglobulins/HRP (DakoCytomation, Glostrup, Denmark) were used as secondary antibodies as appropriate, and HRP-labelled proteins were detected by chemiluminescence (ECL-Amersham, Thermo Fisher Scientific, Loughborough, UK). Band intensities were quantified by Image Lab Software v 5.2.1 (Bio-Rad, Hertfordshire, UK), and data are presented as the ratio of the intensity for the protein of interest/housekeeping protein expressed as a % of the corresponding ratio under control conditions.

### 4.6. Immunoprecipitation

DAPK-1 was immunoprecipitated from treated cell lysates (200 µg total cellular protein) using protein A/G PLUS-Agarose (SANTA CRUZ) as described in the manufacturer’s instructions and in [31]. Briefly, after treatments, cells were lysed in 500 μL cell lysis buffer (10 mM beta glycerophosphate, 1 mM EDTA, 1 mM EGTA, 50 mM Tris-HCl pH 7.5, 1 mM Na orthovanadate, 1 mM benzamidine, 0.2 mM phenyl methyl sulphonyl fluoride, 5 μg/mL of pepstatin A and leupeptin, 1% Triton X-100 and 0.1% beta-mercaptoethanol). To remove any endogenous immunoglobulin from the lysate, samples were precleared with 10 μL of a 50% *v*/*v* protein-A/G PLUS-Agarose suspension for 30 min. The initial sample of protein A/G beads was centrifuged out, and supernatants were then incubated overnight with 12 μL mouse monoclonal antibody against DAPK-1 (Sigma). Then, 10 μL 50% *v*/*v* protein-A/G PLUS-Agarose was added, and incubation was performed for 30 min on ice with shaking. Antibody–antigen complexes were washed four times with the same lysis buffer as above, and SDS-PAGE loading buffer was added. Proteins were separated through SDS-PAGE, and the gel was transferred onto nitrocellulose for Western blotting using antibody against immuno-precipitated (IP) p-DAPK-1 (S308) (Sigma).

### 4.7. GFP-Trap (IP) TPM-3 Coupled Proteomics

Cells expressing TPM-3-Tagged-GFP were treated with MEM containing 1 and 2.5 mM [Pi] for 1.5 h and used subsequently for GFP-Trap^®^ MA (Chromotek, Planegg-Martinsried, Germany) immunoprecipitation of the GFP-TPM-3 fusion protein starting with 200 µg total cell protein, according to the manufacturer’s instruction. Purified TPM-3-GFP was separated on SDS-PAGE followed by either in-gel digestion and proteomics analysis by LC-MS/MS or blotting onto a nitrocellulose membrane.

### 4.8. Determination of the Inhibitory Effect of Pi on PP2A Activity

The inhibitory effect of Pi ions on PP2A catalytic activity was determined in pulled-down PP2A from EA.hy926 cell lysates using a PP2A Immunoprecipitation Phosphatase Assay Kit (Millipore, Burlington, MA, USA) as described in the manufacturer’s instructions. Activity was determined colorimetrically by measuring Pi release from an L-threonine phosphopeptide (K-R-pT-I-R-R).

### 4.9. Confocal Microscopy

Confocal microscopy was used for immunofluorescent visualisation of F-actin. Cells (1000 cells per well) were plated in 8-well slide-chambers (Nunc^®^ Lab-Tek^®^). After treatment with 1 and 2.5 mM [Pi] for 1.5 h, they were fixed with 3.7% formaldehyde and permeablised with 0.1% saponin in PBS, pH 7.5. F-actin was detected using Alexa-Fluor^®^-594-phalloidin (Thermo Fisher Scientific, Loughborough, UK). Nuclei were counterstained by DAPI. The cells were visualised by confocal microscopy using an Olympus FV1000 CLSM. Cells expressing transcytoplasmic actin stress fibres were blind-scored (as previously described [31] with the addition of the frequency distribution of the actin stress fibre scores, calculated from three separate experiments). Briefly, a scoring calibration was set to 0, 0.5, 1, 1.5 or 2, where a score of “0” was given to cells in which all the staining was observed in the mats of stress fibres visible deep inside the cells; a score of “1” was assigned if there were similar amounts of staining in the mats of stress fibres and in the dense cortical actin deposits or in thick dense active fibres; a score of “2” was assigned where nearly all staining was observed in the dense actin deposits on the edge of the cell image or in thick dense actin fibres. Intermediate scorings of 0.5 and 1.5 were also assigned as appropriate.

### 4.10. Statistical Analyses

Unless otherwise stated, data are presented as the mean *+* SEM. Sample size denotes the number of independent experiments. Data were analysed using GraphPad Prism 8.0, and normality was checked with the Kolmogorov–Smirnov test or, if sample size was insufficient, by the Shapiro–Wilk test. Two group data comparisons were analysed by a t test (for normally distributed data) or by Wilcoxon matched-pairs signed rank test (for nonparametric data). One-way ANOVA combined with Tukey’s or Dunnett’s posthoc test was applied for multiple comparison tests as appropriate. Actin stress fibres and their distribution frequency were blind-scored and analysed by nonparametric ANOVA combined with a Chi-square test. *p* values < 0.05 were considered statistically significant.

## Supplementary Materials

Supplementary materials can be found at https://www.mdpi.com/1422-0067/21/19/6993/s1. Supplementary Figure S1. Controls for GFP-TPM3-Trap. (MM) Molecular Marker, (1) GFP-Vector Trap, (2) Flow throw from GFP-Vector Trap, (3) Crude cell lysate from GFP-Vector, (MM) Molecular Marker, (4) GFP-TPM3 Trap, (5) Flow throw from GFP-TPM3 Trap, (6) Crude cell lysate from GFP-TPM3. GFP-Vector Trap and GFP-TPM3 Trap bands from lanes (1) and (4) respectively, were extracted and subjected to proteomic analysis for protein identification. The former was shown to be GFP and the latter TPM3 (Data not shown). (Gel stained with Coomassie brilliant blue). Supplementary Figure S2. Confirmation by sequencing of site-directed mutation of TPM-3 C-terminus from Ser284 (TCT) to pseudophosphorylated Glu284 (GAA). Supplementary Figure S3. Controls for the expression of the wildtype and mutant levels of TPM-3. Cultures transfected with plasmid overexpressing pseudophosphorylated TPM-3 mutant (TPM-3S284E) or empty vector followed by Pi-loading for 1.5 h.
