# Supplementary material for: Inorganic Phosphate (Pi) Signaling in Endothelial Cells: A Molecular Basis for Generation of Endothelial Microvesicles in Uraemic Cardiovascular Disease
Source: Int J Mol Sci. 2020 Sep 23;21(19):6993. doi: 10.3390/ijms21196993 (PMC7583816; doi:10.3390/ijms21196993)
Supplement: Supplementary file 1 [file ijms-21-06993-s001.pdf]

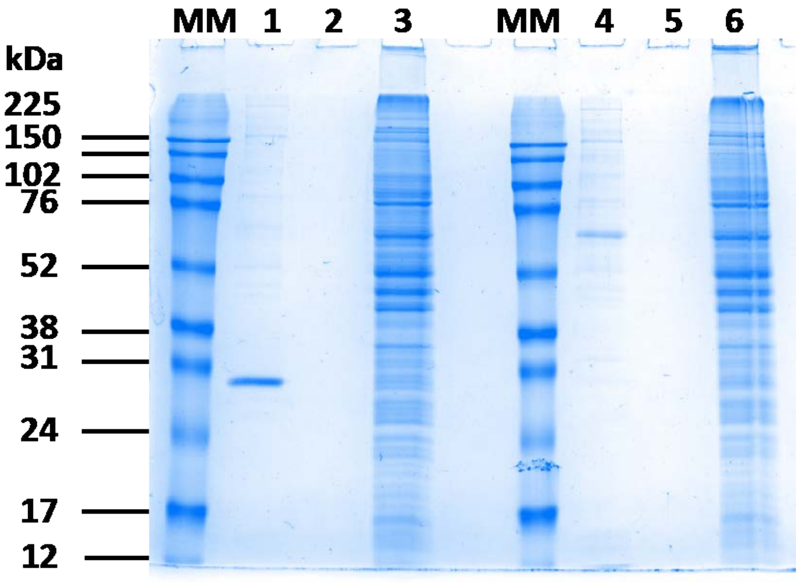

Supplementary Figure S1. Controls for GFP-TPM3-Trap. (MM) Molecular Marker, (1) GFP-Vector Trap, (2) Flow throw from GFP-Vector Trap, (3) Crude cell lysate from GFP-Vector, (MM) Molecular Marker, (4) GFP-TPM3 Trap, (5) Flow throw from GFP-TPM3 Trap, (6) Crude cell lysate from GFP-TPM3. GFP-Vector Trap and GFP-TPM3 Trap bands from lanes (1) and (4) respectively, were extracted and subjected to proteomic analysis for protein identification. The former was shown to be GFP and the latter TPM3 (Data not shown). (Gel stained with Coomassie brilliant blue).

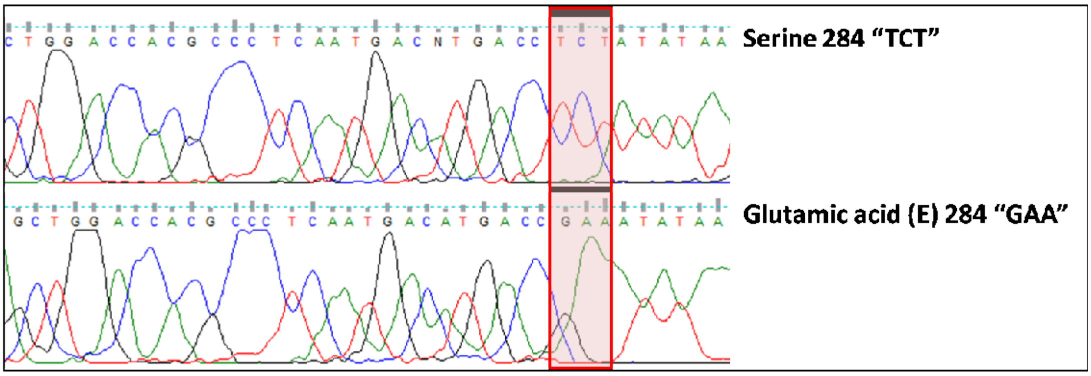

Supplementary Figure S2. Confirmation by sequencing of site-directed mutation of TPM-3 C-terminus from Ser284 (TCT) to pseudophosphorylated Glu284 (GAA).

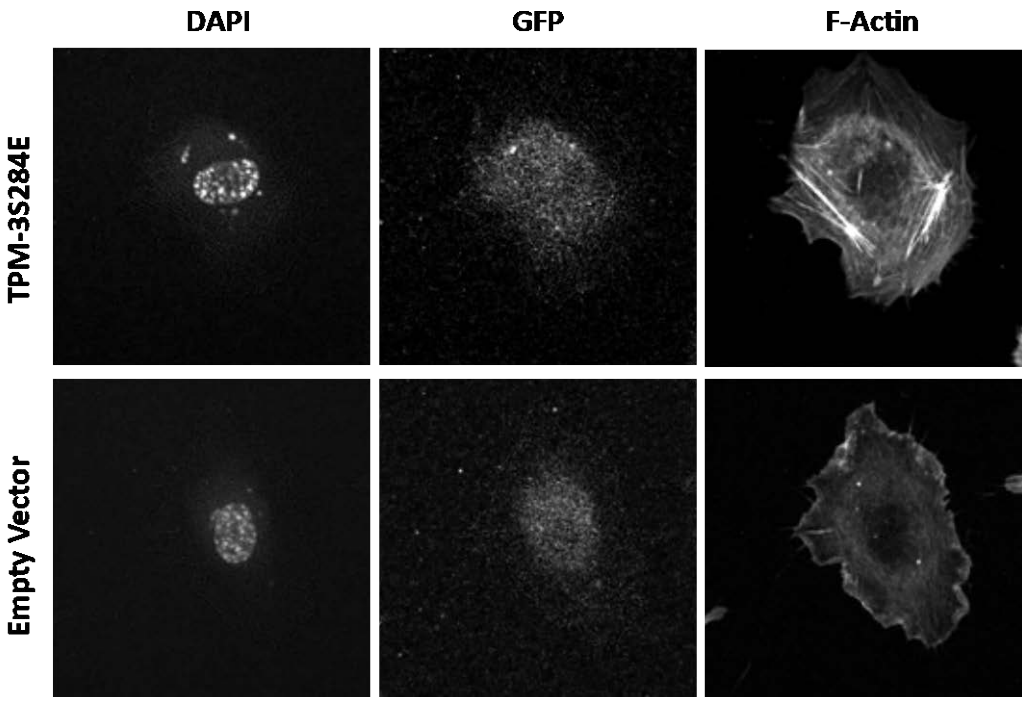

Supplementary Figure S3. Controls for the expression of the wildtype and mutant levels of TPM-3. Cultures transfected with plasmid overexpressing pseudophosphorylated TPM-3 mutant (TPM-3S284E) or empty vector followed by Pi-loading for 1.5h.
